# Draft genome sequence of an *Acinetobacter courvalinii* isolate from Africa

**DOI:** 10.1128/mra.00501-25

**Published:** 2025-07-07

**Authors:** Ahmed Olowo-Okere, E. Skiebe, G. Wilharm

**Affiliations:** 1 Department of Pharmaceutical Microbiology and Biotechnology, Faculty of Pharmaceutical Sciences, University of Abuja 99399, Gwagwalada, Nigeria; 2 Project Group P2, Robert Koch Institute, Wernigerode, Germany; DOE Joint Genome Institute, Berkeley, California, USA

**Keywords:** *Acinetobacter courvalinii*, draft genome, soil, Nigeria

## Abstract

We present the draft genome sequence of an *Acinetobacter courvalinii* isolate from Africa. The strain ABJ_C3_5 was isolated from a Nigerian soil sample and has a 3.76 Mbp genome with a GC content of 43.01%. The genome encodes 3,620 predicted genes, including *adeB*, *adeF*, *ANT(3″)-IIc*, and *bla*_OXA-297_.

## ANNOUNCEMENT

*Acinetobacter courvalinii* was first described in 2016 and has since been isolated from various clinical and environmental sources (1, 2). A colistin-resistant, hypervirulent strain of *A. courvalinii* has also been described (3). However, to date, there have been no reports of *A. courvalinii* from either clinical or environmental sources in Africa. In this study, we sequenced and analyzed the genome of strain ABJ_C3_5, isolated from a flower garden within the University of Abuja, Gwagwalada, Nigeria, in August 2024.

The strain *A. courvalinii* ABJ_C3_5 was isolated from a soil sample (collected at 8°59′03″N, 7°10′16″E) pre-enriched in mineral salts medium supplemented with 0.2% sodium acetate. It was cultured on CHROMagar *Acinetobacter* (without the MDR supplement) and incubated at 37°C. A pure strain was obtained by subsequent serial sub-culturing on Columbia agar supplemented with 5% sheep blood. The genomic DNA of an overnight culture of the strain in Luria-Bertani broth was extracted using the MasterPure DNA Purification Kit (Epicentre) and quantified with a Qubit fluorometer. Sequencing of the genome on an Illumina NextSeq platform (Illumina, Inc., San Diego, CA, USA) generated 8,349,774 paired-end reads (2 × 300 bp). Raw reads were assembled using Shovill version 1.1.0 (https://github.com/tseemann/shovill), with the --trim option and the default SPAdes version 4.0 assembler (4). Genome assembly metrics, contamination, and completeness were determined using QUAST version 5.2.0 and CheckM2 version 1.0.2 (5, 6). Prediction of antibiotic resistance genes was done using ABRicate version 1.0.1 (https://github.com/tseemann/abricate) against the Comprehensive Antibiotic Resistance Database (7, 8). All tools were run with default parameters unless otherwise specified.

The draft assembly comprised 79 contigs, with a total genome size of 3,763,920 bp, a GC content of 43.01%, and an *N*_50_ of 125,597 bp. The completeness and contamination level of the genome were 100% and 0.21%, respectively. Genome annotation was performed using the NCBI Prokaryotic Genome Annotation Pipeline (9), which predicted 3,620 genes, including 3,510 protein-coding sequences, 63 tRNAs, 6 rRNAs, and 6 ncRNAs (Table 1).

**TABLE 1:** Assembly and genome annotation metrics for *Acinetobacter courvalinii* ABJ_C3_5

Metric	Value
No. of reads	8,349,774
Average read length	268
Completeness (%)	100
Contamination (%)	0.21
Total contigs	79
Largest contig (bp)	313,113
Total genome size (bp)	3,763,920
GC content (%)	43.01
*N*_50_ (bp)	125,597
*L*_50_	9
*N*_90_ (bp)	29,135
*L*_90_	37
Total genes	3,620
CDSs (total)	3,545
Coding genes	3,510
CDSs with protein	3,510
Total RNA genes	75
rRNAs	6
tRNAs	63
ncRNAs	4
Pseudogenes	35
Predicted antimicrobial resistance genes	*adeB, adeF, ANT(3″)-IIc, AAC(6′)-Ir, bla*_OXA-297_

**Fig 1:**
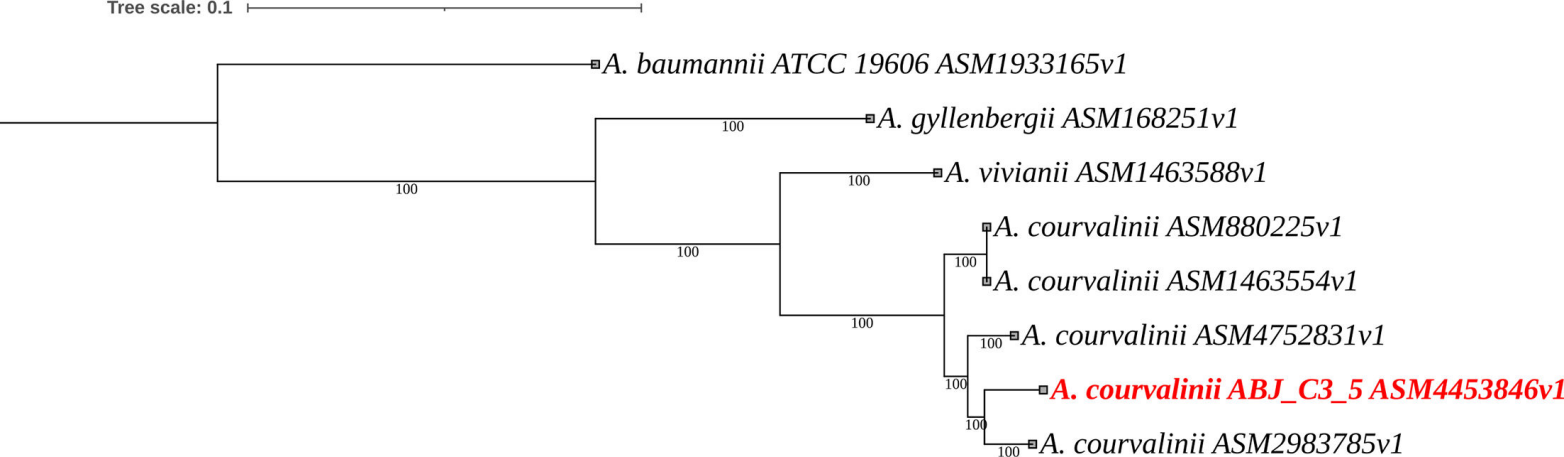
Core-genome phylogenetic tree of *A. courvalinii* strain ABJ_C3_5 and its closest related species

Overall genome relatedness index was computed between the strain ABJ_C3_5 (ASM4453846v1) and the genome of *A. courvalinii* CCM 8635^T^ (ASM1463554v1) using pyani version 0.3.0 (10), EzAAI version 1.2.3 (11), and TYGS (12). Average nucleotide identity (ANI) was calculated at 96.5%, amino acid identity (AAI) at 97%, and digital DNA–DNA hybridization (dDDH) using TYGS formula 2 at 69%. Although the dDDH value falls slightly below the traditional 70% species threshold, both ANI and AAI exceed widely accepted species delineation cutoffs (13). To further establish the taxonomic position of the strain, a core genome phylogenomic tree of the strain and genomes of type strains of closely related *Acinetobacter* spp. was constructed using Panaroo version 1.3.4 (14) for core genome analysis and IQ-TREE version 2.3.6 (15) for maximum likelihood phylogenetic inference. The resulting tree was visualized using Interactive Tree Of Life (https://itol.embl.de). Strain *A. courvalinii* ABJ*_*C3_5 clustered robustly within the *A. courvalinii* clade, strongly confirming its placement within this species despite the marginal dDDH result (Fig. 1)

In addition, the genome harbored several genes associated with antimicrobial resistance, including the multidrug efflux pump genes, *adeB* and *adeF*, aminoglycoside resistance genes, *ANT(3″)-IIc* and *AAC(6′)-Ir,* and the class D beta-lactamase gene, *bla*_OXA-297_.

This study presents the draft genome sequence of an *A. courvalinii* isolate from Africa. The findings contained therein extend the known environmental and geographic range of the species and provide valuable insight into the species’ evolution, resistance mechanisms, and potential for dissemination in the environment.

## Data Availability

The draft genome sequence of strain ABJ_C3_5 has been deposited in GenBank under accession number JBIUCR000000000. Raw reads are available under the BioProject number PRJNA1179717 and SRA accession number SRR33418948.
